# Adaptive Effects of Seeing Green Environment on Psychophysiological Parameters When Walking or Running

**DOI:** 10.3389/fpsyg.2019.00252

**Published:** 2019-02-12

**Authors:** Walid Briki, Lina Majed

**Affiliations:** Sport Science Program, College of Arts and Sciences, Qatar University, Doha, Qatar

**Keywords:** colors, perceived effort, mood states, arousal, pleasure, heart rate, locomotion

## Abstract

Several studies have investigated the influence of perceiving colors on affective outcomes and/or performance. However, the effects of seeing colors on self-selected behaviors have received little attention from physiologists and psychologists. Therefore, the present study aimed at examining whether exposure to green and red environments could influence affective judgments, perception of effort, heart rate, and gait speeds when walking and running at a self-selected pace. Participants were randomly assigned to one of the three experimental conditions: Green, red, or white (neutral) environment. The experimental task consisted in a 20-min trial of either walking (Study 1) or running (Study 2) at the most comfortable speed on a treadmill surrounded by three large HD TV screens displaying specific properties of the studied colors. Study 1 revealed that walking in a green environment induced a significant reduction in heart rate values as compared to the red and white conditions although no differences in gait speed were found. This corroborates the calming and relaxing effect of green on the human organism. Study 2 showed that running in a green environment was associated with an increased level of perceived exertion at similar speeds (compared to other color conditions), while exposure to red induced a significant decrease in the level of tension. In both studies, the preferred gait speed was not affected by the colored environment which is discussed in relation to the energy-conservation principle. Furthermore, both studies showed that performing a 20-min walk or run at preferred pace presented beneficial mood changes. Implications of the effects of self-selected exercise under colored environments on human functioning are addressed in the discussion.

## Introduction

A relatively recent yet growing body of literature is focusing on the psychological, physiological, and behavioral effects of perceiving colors and colored environments. The visual properties of colors seem to hold specific meanings that are centrally processed and thought to influence affects, perceptions, cognitions, as well as intellectual and motor performances (e.g., Elliot et al., 2007; Briki et al., 2015; Briki and Hue, 2016; Krenn, 2018; Lee et al., 2018; Zhang et al., 2018). Early studies on color psychology started on animal species and emphasized the association between physical red coloration and male dominance (e.g., Milinski and Bakker, 1990; Pryke et al., 2002). Such studies suggested that red coloration would underlie a testosterone-related cue reflecting aggressiveness. Hill and Barton (2005) exported such an interest to the field of sport performance by showing a causal relationship between wearing red and winning contests in Olympic combat sports. Subsequent studies supported these findings in real and virtual sporting contexts (e.g., Attrill et al., 2008; Ilie et al., 2008).

Consequently, a central question raised the concern on the origin of the effect of red on competitive outcomes. Indeed, the results of Hill and Barton’s study did not allow to know if “wearing red” or “perceiving red” was responsible for behavior changes in competitive situations. Therefore, two research directions emerged focusing either on actors wearing red (e.g., Ten Velden et al., 2012; Dreiskaemper et al., 2013; Wiedemann et al., 2015) or on observers perceiving red (e.g., Hagemann et al., 2008). Studies exploring the effects of wearing red evidenced the beneficial effects of red on actors in terms of confidence and competitiveness. For example, a study revealed that participants wearing red had significantly higher heart rate values as compared to those who were wearing blue and developed greater leg strength compared to those wearing blue and other colors (Dreiskaemper et al., 2013). Other studies found that poker players using red poker chips made more risky decisions than using blue poker chips (Ten Velden et al., 2012) or that people wearing red were viewed as being more dominant (Wiedemann et al., 2015). Regarding the studies conducted on observers, research indicated that red impaired intellectual performance (e.g., Elliot et al., 2007) and endurance motor performance (Briki et al., 2015), however, improved forceful and explosive motor performance (Elliot and Aarts, 2011). The authors consistently explained their findings by suggesting that perceiving red would stimulate the avoidance motivational system, which would mobilize energy to attain defensive purposes. Such an avoidance-based functioning would be responsible for the above-mentioned positive (for the forceful tasks) and negative (for the intellectual and endurance-related motor tasks) effects on performance (Elliot and Maier, 2014).

The present study focused on the effects of perceiving colors on affective, physiological, perceptual and behavioral responses. Its originality was to explore the influence of red and green environments on self-selected behaviors, with no performance-oriented goal. Considered as the color of natural and forestial environments, green is often associated with positive meanings and representations (e.g., tree, oxygen, breath, life) (Barton et al., 2012, 2016; Lee et al., 2018). Studies showed that green natural environments generated therapeutic and positive effects, such as fostering recovery from surgery (Ulrich, 1984) and subjective well-being (Kaplan, 2001; van den Berg et al., 2003). Studies focused on exercise under controlled laboratory environments also revealed that perceiving green enhanced positive affective and cognitive outcomes (e.g., enjoyment, self-esteem, motivation) and diminished negative ones (e.g., mood disturbance, anxiety) (e.g., Akers et al., 2012; Barton et al., 2012; Briki et al., 2015; Briki and Hue, 2016). Briki and Hue (2016) exposed participants to colored laptop screens and observed that: (a) arousal was higher while seeing green than while seeing white; and (b) seeing green was more pleasurable than seeing red. Briki et al. (2015) confronted athletes with different colored computed screens while achieving series of three randomized 7-min trials on home trainers. The authors showed that: (a) covered distance and heart rate were higher within the gray and green environments than within the red environment; (b) enjoyment was higher within the green environment than within the red environment; and (c) perceived effort and anxiety were not influenced by the colored environments. Using cycling tasks at a controlled pace (i.e., moderate intensity) in front of edited nature-related videos of different colors (green, red, and gray) displayed by a screen, his Akers et al. (2012) showed that participants exposed to the green-forestial condition displayed lower levels of perceived exertion, total mood disturbance and anger as compared to those who were exposed to red- and gray-forestial conditions. However, the authors did not find differences between the color conditions for physiological responses (i.e., oxygen consumption and heart rate). In sum, the studies suggest that exposure to green environmental cues could increase the occurrence of agreeable and adaptive outcomes, and this would be due to the activation of the approach motivational system (Elliot and Maier, 2007, 2014; Elliot et al., 2009), reputed to often promote positive and adaptive responses (e.g., Elliot et al., 2011; Mascret et al., 2015).

From a motor control perspective, the adoption of preferred behaviors is believed to reflect the tendency of the human musculoskeletal system to minimize the many cost functions, such as the metabolic energy cost (Sparrow and Newell, 1998). Although humans do not always behave in an energy-saving mode (e.g., imposed exercise intensity), most of our behaviors are learned by trial and error and governed by energy-saving principles (e.g., Alexander, 2002; Gendolla and Richter, 2010). The two main modes of progression are fashioned through long phylogenic refinements, allowing humans to either displace at slow speeds/intensities for long periods of time (i.e., walking) or at high speeds/intensities for short periods of time (i.e., running). The dynamical perspective of human locomotion considers walking and running as two stable behaviors (i.e., attractors), which are thought to organize as a result of complex interactions taking place among the multiple individual, environmental and task-related constraints (e.g., Newell, 1986). From a dynamical perspective, a constraint reflects a parameter that is likely to foster the emergence of specific responses. For example, speed was employed as a constraint to examine the natural tendencies of the locomotor system, and it was found that a gradual increase in speed led the locomotor system to abruptly transit from a walking to a running pattern, and vice-versa in response to a decrease in speed (e.g., Diedrich and Warren, 1995; Raynor et al., 2002; Ganley et al., 2011). Interestingly, research also found that changes in optic flows could modulate the walk-to-run transition (De Smet et al., 2009), pointing at the potential influence of visual stimuli on the adoption of preferred gait patterns.

As opposed to numerous classic color psychology studies–placing emphasize on performance–, the present research aimed at studying the effects of exposure to green and red environments when freely selecting a walking or running pace. How could the combination between exposure to colored environment and preferred gait patterns influence the physiological, affective, cognitive, and behavioral outcomes? In this research, we argue that exposure to a colored environment can play the role of an environmental constraint that can contribute to the precipitation of specific physiological, psychological, and behavioral responses. Indeed, we presume that the interactions between the visual properties of the environment (e.g., color green) and the task itself (e.g., walking or running) would induce specific affective, physiological, and behavioral responses. Because green (or red) is presumed to activate approach-based (or avoidance-based) regulation, we expect that exposure to green (or red) while freely walking or running would entail adaptive/facilitative (or maladaptive/debilitative) outcomes. Consistent with the view that our behaviors are driven by the energy-conservation principle (e.g., Gendolla and Richter, 2010), we hypothesize that exposure to green would promote the tendency of human organism to preserve its energy, thereby leading to a “slowing down” when walking or running. By contrast, exposure to red would debilitate such a functioning, thus leading to elevate gait speed and reduce physiological efficiency. The present investigation included two studies performed on a motorized treadmill surrounded by three large HD screens displaying specific properties of red, green and white. Study 1 consisted in walking at self-selected speeds for 20 min and Study 2 involved running at self-selected speeds for the same period of time. Based on the above-mentioned rationale, we specifically expect in both studies that exercising in a green environment, as compared to a white one, would be associated with greater levels of positive affective states and lower levels of negative affective states, perceived effort, heart rate, and gait speed. We also expect to observe opposite results for the red condition, as compared to the white condition.

## Study 1

### Methods

#### Participants

Twenty-nine young female adults (*M*_age_ = 22.34 years, *SD*_age_ = 1.76; *M*_height_ = 1.60 m, *SD*_height_ = 0.06; *M*_weight_ = 60.50 kg, *SD*_weight_ = 13.82) volunteered to participate in the study. Participants were healthy and presented no evidence of past or present metabolic, cardiovascular, neuromuscular or musculoskeletal dysfunctions or injuries. Participants presenting any other health problem that might interfere with exercise or an inability to walk or run continuously on the treadmill were excluded from the study. The final sample was composed 26 participants (*M*_age_ = 22.35 years, *SD*_age_ = 1.76; *M*_height_ = 1.59 m, *SD*_height_ = 6.57; *M*_weight_ = 60.64 kg, *SD*_weight_ = 14.50) as they reported acceptable levels of perceived color typicality (see section “Measures” below). Participants were then randomly assigned to one of three experimental groups consisting of three color conditions (green: *n* = 8; red: *n* = 8; white: *n* = 10). All volunteers were asked to bring comfortable sports outfit and shoes to the laboratory for the experimental procedures. Prior to the experimental sessions, all participants signed a written informed consent in accordance to the Declaration of Helsinki. The experimental procedures were approved by the Institutional Review Board of Qatar University (IRB: QU-IRB 573-E/16).

#### Materials

The experiment was performed on a motorized treadmill with side handrails, characterized by a walking surface of 60 × 170 cm^2^ and a speed range of 1–25 km.h^-1^ (Valiant 2 CPET, Lode, Netherlands). Three identical 58 inch HD TV screens (Hisense, Smart Ultra HD 4K LED, China) surrounded the treadmill from the front and anterior left and right sides and were positioned at a height that aligns their mid-vertical distance with the participants’ eye-level. All screens displayed the visual properties of one of the three colors created using the hue-saturation-value (hsv) color model, with red (0°, 0%, 0%), green (120°, 100%, 100%), and white (0°, 0%, 100%). Heart rate was measured using a polar belt wrapped around the chest (Polar, Kempele, Finland).

#### Design and Procedure

The study comprised three distinct phases performed on the same visit to the Sport Science Laboratory: Pre-experiment (20 min), experiment (20 min), and recovery (10–15 min).

##### Pre-experiment phase

Prior to experiments, participants’ anthropometric measurements (i.e., body weight, body height, and leg length) were assessed, after which a pre-experiment mood state questionnaire was filled. Participants were then instructed and trained on how to report their scores on psychological scales (i.e., pleasure, arousal, and perceived exertion) that would appear on the front screen. Lastly, in order to warm-up and familiarize with the treadmill, participants were asked to start a 7-min walk at a preferred speed in a neutral environment (white) followed by a 3-min rest.

##### Experiment phase

During the experiment period, participants were asked to walk for 20 min on the treadmill at their most comfortable speed. They were informed that: (a) the 20-min trial would be divided into four continuous 5-min intervals; (b) at the beginning of each 5-min interval, a 30-s period would be given to adjust the speed to maintain the most comfortable pattern (if needed); and (c) at the end of each 5-min interval, a 30-s period would be given to report verbally their score of perceived exertion, pleasure, and arousal (10 s per question) displayed on the front screen. Importantly, the participants were blind to the speed displayed on the treadmill. Finally, in order to reinforce their immersion in the colored environment, the experimental task was performed in a totally dark room where the only light source came from the diffused color displayed on the three screens.

##### Recovery phase

During the recovery period, participants were invited to rest during 5 min, after which they answered the post-experiment mood state questionnaire (i.e., recovery). Finally, participants were asked to rate their perception of color typicality.

#### Measures

The measures consisted in collecting psychological, physiological, anthropometric, and preferred walking speed. While certain measures aimed to test the hypotheses (i.e., main measures), others aimed to control the quality of the experimental design and to reinforce the reliability of the results (i.e., control measures).

##### Main measures

Self-selected gait speed corresponded to the value of treadmill speed collected by the experimenter at each interval. Heart rate was measured continuously throughout the test and the dependent variable corresponded to the mean heart rate value collected during the last minute of each 5-min interval (to ensure that a steady-state was reached). Scores of perceived exertion were collected using a printed 15-point Borg Rating of Perceived Exertion Scale, ranging from 6 to 20 (Borg, 1973). The ratings of pleasure and arousal were collected using the Self-Assessment Manikin (Bradley and Lang, 1994). Arousal corresponds to the notion of physiological activation, from a wide-eyed and excited image to a sleepy and relaxed image. The words “excited,” “nervous,” and “wide-awake” were used to describe the extreme left of the scale (coded by the score 5), whereas the words “calm,” “relaxed,” and “sleepy” were used to describe the extreme right of the scale (coded by the score 1). Pleasure corresponds to the affective valence, from a positive image to a negative image. The words “positive,” “pleasant,” and “happy” were used to describe the extreme left of the scale (coded by the score 1), whereas the words “negative,” “unpleasant,” and “unhappy” were used to describe the extreme right of the scale (coded by the score 5). The mood states were assessed using the abbreviated version of the Profile of Mood States (POMS) questionnaire, rated on a 5-point Likert scale (0 = “*Not at all*,” 1 = “*A little*,” 2 = “*Moderately*,” 3 = “*Quite a lot*,” 4 = “*Extremely*”) (Grove and Prapavessis, 1992). The POMS encompassed the subscales of tension, depression, anger, vigor, fatigue, confusion, and esteem-related affect. Total mood disturbance was a complementary subscale resulting from an equation including all constructs, as follows: Total mood disturbance = [(tension + depression + anger + fatigue + confusion) – (vigor + esteem)] + 100.

##### Control measures

We assessed anthropometric variables, such as body height, body weight, and leg length (i.e., vertical distance from the greater trochanter of the right femur to the standing surface). Furthermore, Elliot and Maier (2014) it was suggested that “…equating colors on perceived typicality bolsters the rigor of empirical work on color” (p. 98). Therefore, and consistent with what authors did (e.g., Elliot et al., 2007), we controlled perceived color typicality. The scores of 3, 4 and 5 were considered acceptable to the question “To what degree is the color [the name of the assigned color] of the environment a typical example of that color?,” rated on a Likert scale from 1 (“*Not at all*”) to 5 (*Very much so*).

#### Data Analysis

Preliminary one-way ANOVAs were conducted to examine possible differences across the three independent experimental groups, in terms of anthropometric characteristics and perceived color typicality. Furthermore, to test our hypotheses, 3 × 4 repeated measures ANOVAs (Colored Environment: Green, red, white × Time Interval: 0–5, 5–10, 10–15, and 15–20 min) were performed on pleasure, arousal, perceived effort, heart rate, and self-selected gait speed. In addition, 3 × 2 repeated measures ANOVAs (Colored Environment: Green, red, white × Experimental Phase: Pre-experiment and recovery) were performed on mood states (i.e., tension, depression, anger, vigor, fatigue, confusion, esteem-related affect, and total mood disturbance). When needed, the analyses were completed by *post hoc* LSD tests for pairwise comparisons. Each repeated measures ANOVA was preceded by a Mauchly’s sphericity test, and when the test was significant (indicating a violation of the hypothesis of variance homogeneity) a Huynh-Feldt correction procedure was used to adjust the degrees of freedom. All statistical tests were conducted with IBM SPSS Statistics (version 24), with a level of significance set at *p* < 0.05.

### Results and Discussion

#### Preliminary Results

The analyses revealed no significant main effect of Colored Environment on perceived color typicality, body height, body weight, and leg length (see Tables 1, 2). Therefore, the main findings of the present study may more likely be attributed to the experimental conditions than to any group-based differences.

**Table 1:** Results of ANOVAs on control (typicality and anthropometric variables) and main measures (self-selected gait speed, heart rate, perceived exertion, pleasure and arousal).

		Study 1: walking			Study 2: running		
Variable	Effect	*F*	*p*	*$\eta_{p}^{2}$*	*F*	*p*	*$\eta_{p}^{2}$*
Typicality	Colored environment	(2,23) = 1.97	0.16	0.15	(2,25) = 2.00	0.16	0.14
Body height	Colored environment	(2,23) = 0.43	0.65	0.04	(2,25) = 0.63	0.54	0.05
Body weight	Colored environment	(2,23) = 0.13	0.88	0.01	(2,25) = 0.21	0.81	0.02
Leg length	Colored environment	(2,23) = 0.38	0.69	0.03	(2,25) = 0.08	0.92	0.01
Self-selected gait speed	Colored environment	(2,23) = 1.10	0.35	0.09	(2,25) = 0.07	0.93	0.01
	Time interval	(1.52,35.01) = 12.77	0.00	0.36	(2.33,58.28) = 0.66	0.54	0.26
	Colored environment × Time interval	(3.05,35.01) = 1.34	0.28	0.10	(4.66,58.28) = 0.68	0.63	0.05
Heart rate	Colored environment	(2,23) = 3.70	0.04	0.24	(2,25) = 1.94	0.16	0.14
	Time interval	(1.86,42.72) = 4.34	0.21	0.16	(1.84,46.02) = 3.16	0.06	0.11
	Colored environment × Time interval	(3.71,35.01) = 1.39	0.26	0.10	(3.68, 46.02) = 0.55	0.69	0.04
Perceived exertion	Colored environment	(2,23) = 0.58	0.57	0.05	(2,25) = 0.65	0.53	0.05
	Time interval	(2.28,52.32) = 7.60	0.00	0.25	(2.25,56.21) = 4.83	0.01	0.16
	Colored environment × Time interval	(4.55,35.01) = 1.60	0.18	0.12	(4.50, 56.21) = 2.62	0.04	0.17
Pleasure	Colored environment	(2,23) = 0.04	0.96	0.00	(2,25) = 1.25	0.31	0.09
	Time interval	(2.73,62.84) = 1.18	0.32	0.05	(3,75) = 1.32	0.28	0.05
	Colored environment × Time interval	(5.46,35.01) = 1.19	0.32	0.09	(6,75) = 1.47	0.20	0.11
Arousal	Colored environment	(2,23) = 0.50	0.61	0.04	(2,25) = 0.94	0.40	0.07
	Time interval	(2.79,64.12) = 0.30	0.81	0.01	(2.27,56.66) = 1.43	0.25	0.05
	Colored environment × Time interval	(5.58,35.01) = 0.21	0.97	0.02	(4.53, 56.66) = 0.89	0.49	0.07

**Table 2:** Mean scores (M) and standard deviations (SD) or standard errors (SE) of self-selected gait speed (km.h^-1^), heart rate (beat.min^-1^), perceived effort, pleasure, and arousal as a function of Colored Environment and Time Interval.

		Study 1: walking				Study 2: running			
		Green	Red	White	All	Green	Red	White	All
Variable	Interval	*M (SD) [SE]*	*M (SD) [SE]*	*M (SD) [SE]*	*M (SD) [SE]*	*M (SD) [SE]*	*M (SD) [SE]*	*M (SD) [SE]*	*M (SD) [SE]*
Typicality	–	4.62 (.52)	4.00 (.76)	4.50 (.71)	4.63 (0.52)	4.10 (.88)	3.67 (.71)	4.44 (.88)	4.07 (0.86)
Body H.	–	158.19 (5.10)	158.37 (6.52)	160.80 (7.90)	4.00 (0.76)	162.50 (5.23)	161.22 (3.38)	164.11 (7.16)	162.61 (5.40)
Body W.	–	58.50 (7.16)	62.19 (14.00)	61.12 (19.64)	4.50 (0.71)	60.80 (9.87)	60.66 (9.43)	64.13 (17.84)	61.83 (12.48)
Leg length	–	91.06 (2.70)	89.94 (3.99)	91.80 (5.78)	4.38 (0.70)	93.75 (4.55)	92.93 (2.51)	93.39 (5.66)	93.37 (4.29)
Self-selected gait speed	0–5 min	3.29 (1.06)	3.94 (1.51)	3.68 (1.16)	3.64 (1.23)	6.86 (1.13)	6.76 (0.55)	6.66 (1.77)	6.76 (1.20)
	5–10 min	3.54 (1.10)	4.53 (1.01)	4.20 (1.25)	4.10 (1.16)	6.42 (1.43)	6.90 (1.26)	6.51 (1.44)	6.60 (1.34)
	10–15 min	3.73 (1.28)	4.48 (0.84)	4.66 (1.50)	4.32 (1.28)	6.18 (1.12)	6.53 (1.06)	6.73 (1.63)	6.47 (1.26)
	15–20 min	3.87 (1.41)	4.50 (0.74)	4.83 (1.68)	4.43 (1.38)	6.54 (1.05)	6.54 (1.21)	6.62 (1.44)	6.57 (1.19)
	All	3.60 [0.42]	4.36 [.42]	4.34 [0.38]	4.10 [0.23]	6.50 [0.35]	6.68 [0.37]	6.63 [0.37]	6.61 [0.21]
Heart rate	0–5 min	104.40 (11.85)	113.56 (12.67)	109.44 (9.14)	109.16 (11.30)	160.83 (30.67)	177.04 (16.76)	173.40 (18.28)	170.08 (23.39)
	5–10 min	94.56 (35.03)	118.01 (9.61)	116.08 (6.19)	110.05 (22.25)	169.16 (18.80)	184.83 (14.58)	177.31 (13.81)	176.82 (16.75)
	10–15 min	108.24 (13.95)	118.64 (11.49)	120.55 (9.98)	116.17 (12.53)	167.36 (21.72)	180.94 (13.85)	181.36 (13.80)	176.23 (17.77)
	15–20 min	112.08 (15.96)	118.40 (13.57)	124.43 (9.40)	118.78 (13.48)	170.00 (23.28)	179.76 (13.79)	182.51 (13.29)	177.16 (17.89)
	All	104.82 [3.86]	117.15 [3.86]	117.63 [3.45]	113.20 [2.15]	166.84 [5.25]	180.64 [5.54]	178.65 [5.54]	175.38 [3.14]
Perceived effort	0–5 min	8.38 (2.13)	8.38 (3.07)	7.30 (2.06)	7.96 (2.39)	10.50 (2.68)	10.56 (1.94)	11.22 (3.56)	10.75 (2.72)
	5–10 min	9.38 (2.20)	7.75 (2.55)	7.70 (1.89)	8.23 (2.25)	11.30 (2.58)	11.44 (3.17)	10.67 (2.00)	11.14 (2.55)
	10–15 min	9.75 (2.38)	8.63 (2.20)	8.90 (2.60)	9.08 (2.37)	13.00 (2.91)	11.33 (2.12)	11.44 (1.94)	11.96 (2.43)
	15–20 min	10.00 (2.56)	8.63 (1.92)	9.80 (3.08)	9.50 (2.58)	13.90 (3.04)	11.00 (2.87)	11.67 (2.50)	12.25 (3.00)
	All	9.38 [0.77]	8.34 [.77]	8.43 [0.68]	8.72 [0.43]	12.18 [0.72]	11.08 [0.76]	11.25 [0.76]	11.50 [0.43]
Pleasure	0–5 min	3.75 (0.707)	4.00 (0.93)	3.50 (1.35)	3.73 (1.04)	4.20 (0.63)	3.89 (1.05)	4.00 (0.87)	4.04 (0.84)
	5–10 min	3.75 (0.89)	4.00 (1.07)	3.70 (1.34)	3.81 (1.10)	3.40 (1.43)	4.00 (1.00)	4.33 (0.50)	3.89 (1.10)
	10–15 min	3.75 (1.04)	3.38 (0.74)	3.50 (1.18)	3.54 (0.99)	3.30 (1.16)	3.56 (1.24)	4.22 (0.83)	3.68 (1.12)
	15–20 min	3.50 (1.20)	3.63 (0.74)	3.80 (1.23)	3.65 (1.06)	3.30 (1.16)	3.89 (0.93)	3.89 (1.05)	3.68 (1.06)
	All	3.69 [0.34]	3.75 [0.34]	3.63 [0.30]	3.69 [0.19]	3.55 [0.25]	3.83 [0.26]	4.11 [0.26]	3.83 [0.15]
Arousal	0–5 min	2.75 (0.87)	2.13 (0.99)	2.60 (1.51)	2.50 (1.18)	3.30 (0.95)	2.56 (0.88)	2.56 (0.88)	2.82 (0.95)
	5–10 min	2.88 (1.25)	2.38 (1.30)	2.50 (1.51)	2.58 (1.33)	3.60 (1.08)	3.11 (1.05)	2.89 (1.17)	3.21 (1.10)
	10–15 min	2.88 (0.99)	2.25 (1.04)	2.80 (1.48)	2.65 (1.20)	3.20 (1.03)	3.00 (1.58)	2.89 (1.17)	3.04 (1.23)
	15–20 min	2.75 (1.17)	2.25 (1.17)	2.50 (1.51)	2.50 (1.27)	3.10 (1.20)	2.44 (0.88)	3.11 (1.36)	2.89 (1.17)
	All	2.81 [0.40]	2.25 [0.40]	2.60 [0.36]	2.55 [0.22]	3.30 [0.28]	2.78 [0.30]	2.86 [0.30]	2.98 [0.17]

*“Body H.” means body height, and “Body W.” means body weight.*

#### Effects of Colors

**FIGURE 1:**
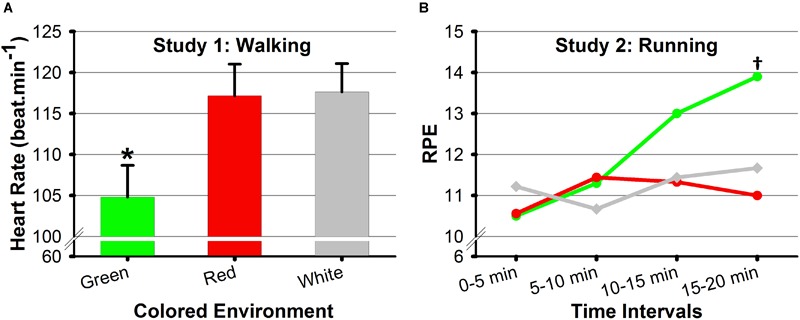
**(A)** Mean scores and standard errors of heart rate (beat.min^-1^) as a function of Colored Environment (Study 1, walking), and **(B)** mean ratings of perceived exertion (RPE) as a function of Colored Environment and Time Interval (Study 2, running). ^∗^Significant difference compared to the red and white conditions, and ^†^significant difference compared to the red condition at a specific time interval.

The analyses indicated that a significant main effect of Colored Environment was found on heart rate (see Table 1), whereas no such main effect was revealed for self-selected gait speed, perceived effort, pleasure, and arousal (see Tables 1, 2). *Post hoc* comparisons showed that for similar walking speeds, heart rate values were lower only in the green environment compared to the white and red ones, *p*s ≤ 0.03 (see Figure 1A and Table 2). This result suggests that seeing green while walking, as compared to seeing red and white, can reduce the physiological activation of the organism. As opposed to the present study, Akers et al. (2012) have failed to show a significant effect of colored environment on cycling heart rate. We believe that this difference might be due to the nature of imposed (i.e., 50% peak power output, Akers et al., 2012) versus self-selected intensities (i.e., present study). Although the literature has distinguished the effects of wearing red and seeing red, to our knowledge this distinction was not yet addressed for the color green. Therefore, our result could relate to Dreiskaemper et al. (2013) study showing that wearing blue–a color reputed to be related to comparable effects as green (see Briki and Hue, 2016)–was associated with lower levels of heart rate, as compared to the wearing red. However, the study’s results could not tell us whether such a change in heart rate resulted from wearing (or seeing) red or blue since the authors did not employ a neutral condition during the ongoing experimental task (i.e., combat) (Dreiskaemper et al., 2013). Our result also supports our prediction that seeing green can entail relaxing effects (e.g., Briki and Hue, 2016) and can promote the preservation of energy. Furthermore, the analyses revealed a Colored Environment × Experimental Phase interaction effect for vigor (see Table 3). *Post hoc* comparisons showed that in the white (neutral) condition vigor was higher after the 20-min effort than before, *p* = 0.02 (see Table 4). This result suggests that the simple fact of walking during 20 min at a self-selected pace can elicit benefits on mood states. This interpretation supports in part studies showing that volitional walking–performed either in a laboratory setting, in a urban environment, or in a natural environment–could induce greater levels of positive feelings and affective judgments (e.g., Ekkekakis et al., 2000; Focht, 2009; Kinnafick and Thøgersen-Ntoumani, 2014; Black et al., 2015). The analyses indicated no additional interaction effects for any variables (see Tables 1, 3).

**Table 3:** Results of ANOVAs (3 Colored Environments × 2 Experimental Phases) on tension, depression, anger, vigor, fatigue, confusion, esteem, and total mood disturbance.

		Study 1: walking			Study 2: running		
Variable	Effect	*F*	*p*	*$\eta_{p}^{2}$*	*F*	*p*	*$\eta_{p}^{2}$*
Tension	Colored environment	(2,23) = 2.01	0.82	0.02	(2,25) = 0.64	0.54	0.05
	Experimental phase	(1,23) = 0.26	0.62	0.01	(2,25) = 11.60	0.00	0.32
	Colored environment × Experimental phase	(2,23) = 0.47	0.63	0.04	(2,25) = 3.35	0.05	0.21
Depression	Colored environment	(2,23) = 0.18	0.83	0.02	(2,25) = 0.81	0.46	0.06
	Experimental phase	(1,23) = 0.99	0.33	0.04	(2,25) = 4.80	0.04	0.16
	Colored environment × Experimental phase	(2,23) = 1.13	0.34	0.09	(2,25) = 2.12	0.14	0.15
Anger	Colored environment	(2,23) = 0.06	0.94	0.01	(2,25) = 1.84	0.18	0.13
	Experimental phase	(1,23) = 0.95	0.34	0.04	(2,25) = 0.60	0.45	0.02
	Colored environment × Experimental phase	(2,23) = 0.63	0.54	0.05	(2,25) = 0.24	0.79	0.02
Vigor	Colored environment	(2,23) = 3.90	0.03	0.25	(2,25) = 0.36	0.70	0.03
	Experimental phase	(1,23) = 0.42	0.53	0.02	(2,25) = 8.16	0.01	0.25
	Colored environment × Experimental phase	(2,23) = 3.90	0.03	0.25	(2,25) = 1.79	0.19	0.13
Fatigue	Colored environment	(2,23) = 0.68	0.52	0.06	(2,25) = 0.51	0.61	0.04
	Experimental phase	(1,23) = 0.12	0.73	0.01	(2,25) = 5.19	0.03	0.17
	Colored environment × Experimental phase	(2,23) = 1.79	0.19	0.13	(2,25) = 0.50	0.61	0.04
Confusion	Colored environment	(2,23) = 2.03	0.15	0.15	(2,25) = 0.89	0.42	0.07
	Experimental phase	(1,23) = 0.92	0.35	0.04	(2,25) = 39.13	0.00	0.61
	Colored environment × Experimental phase	(2,23) = 0.77	0.48	0.06	(2,25) = 2.57	0.10	0.17
Esteem	Colored environment	(2,23) = 0.27	0.76	0.02	(2,25) = 1.55	0.23	0.11
	Experimental phase	(1,23) = 0.03	0.88	0.00	(2,25) = 13.54	0.00	0.35
	Colored environment × Experimental phase	(2,23) = 0.49	0.62	0.04	(2,25) = 1.68	0.21	0.12
Total mood disturbance	Colored environment	(2,23) = 0.18	0.83	0.02	(2,25) = 0.46	0.64	0.04
	Experimental phase	(1,23) = 0.54	0.47	0.02	(2,25) = 12.52	0.00	0.33
	Colored environment × Experimental phase	(2,23) = 2.75	0.09	0.19	(2,25) = 2.49	0.10	0.17

**Table 4:** Mean scores (M) and standard deviations (SD) or standard errors (SE) of tension, depression, anger, vigor, fatigue, confusion, esteem, and total mood disturbance as a function of Colored Environment and Experimental Phase.

		Study 1: walking				Study 2: running			
		Green	Red	White	All	Green	Red	White	All
Variable	Phase	*M (SD) [SE]*	*M (SD) [SE]*	*M (SD) [SE]*	*M (SD) [SE]*	*M (SD) [SE]*	*M (SD) [SE]*	*M (SD) [SE]*	*M (SD) [SE]*
Tension	Pre-test	0.65 (0.58)	0.58 (0.45)	0.75 (0.77)	0.67 (0.61)	0.29 (0.30)	0.78 (0.91)	0.58 (0.67)	0.54 (0.67)
	Post-test	0.90 (0.91)	0.55 (0.62)	0.73 (1.10)	0.73 (0.89)	0.20 (0.30)	0.15 (0.28)	0.38 (0.67)	0.24 (0.44)
	All	0.78 [0.26]	0.56 [0.25]	0.74 [0.23]	0.69 [0.14]	0.24 [0.16]	0.47 [0.17]	0.48 [0.17]	0.40 [0.10]
Depression	Pre-test	0.09 (0.11)	0.36 (0.39)	0.39 (0.61)	0.29 (0.44)	0.11 (0.18)	0.45 (0.74)	0.25 (0.50)	0.27 (0.52)
	Post-test	0.41 (0.90)	0.46 (0.60)	0.30 (0.71)	0.39 (0.71)	0.03 (0.06)	0.12 (0.28)	0.24 (0.48)	0.13 (0.32)
	All	0.25 [0.19]	0.41 [0.19]	0.35 [0.17]	0.34 [0.11]	0.07 [0.13]	0.29 [0.13]	0.25 [0.13]	0.20 [0.08]
Anger	Pre-test	0.33 (0.28)	0.48 (0.51)	0.56 (0.85)	0.47 (0.60)	0.07 (0.21)	0.62 (0.96)	0.28 (0.67)	0.31 (0.69)
	Post-test	0.60 (1.20)	0.73 (1.06)	0.48 (0.98)	0.59 (1.04)	0.07 (0.14)	0.66 (0.89)	0.39 (0.73)	0.36 (0.68)
	All	0.47 [0.28]	0.60 [0.28]	0.52 [0.25]	0.53 [0.16]	0.07 [0.20]	0.64 [0.21]	0.33 [0.21]	0.35 [0.12]
Vigor	Pre-test	2.15 (0.86)	2.28 (1.06)	1.90 (0.94)	2.09 (0.93)	2.24 (0.98)	2.20 (1.01)	2.18 (1.03)	2.21 (0.97)
	Post-test	1.85 (1.22)	2.33 (0.80)	2.38 (0.86)	2.20 (0.96)	2.33 (0.82)	2.68 (0.65)	3.02 (0.88)	2.67 (0.81)
	All	2.00 [0.32]	2.30 [0.32]	2.14 [0.29]	2.15 [0.18]	2.29 [0.25]	2.44 [0.27]	2.60 [0.27]	2.44 [0.15]
Fatigue	Pre-test	1.18 (0.75)	1.23 (0.68)	1.20 (0.81)	1.20 (0.72)	0.56 (0.80)	1.02 (0.77)	0.78 (0.50)	0.78 (0.71)
	Post-test	1.45 (0.94)	1.50 (0.87)	0.83 (0.82)	1.23 (0.90)	1.02 (0.70)	1.18 (0.86)	1.04 (0.88)	1.08 (0.78)
	All	1.31 [0.25]	1.36 [0.25]	1.01 [0.22]	1.23 [0.14]	0.79 [0.22]	1.10 [0.23]	0.91 [0.23]	0.93 [0.13]
Confusion	Pre-test	1.53 (0.80)	1.03 (0.47)	0.73 (0.84)	1.06 (0.78)	0.62 (0.56)	1.08 (0.58)	0.78 (0.67)	0.82 (0.61)
	Post-test	1.25 (0.86)	1.08 (0.67)	0.65 (0.91)	0.97 (0.83)	0.29 (0.40)	0.50 (0.54)	0.53 (0.66)	0.44 (0.53)
	All	1.39 [0.26]	1.05 [0.26]	0.69 [0.23]	1.04 [0.15]	0.46 [0.17]	0.79 [0.18]	0.66 [0.18]	0.63 [0.10]
Esteem	Pre-test	2.22 (1.14)	2.53 (0.88)	2.25 (0.97)	2.33 (0.97)	2.31 (1.00)	2.25 (1.00)	2.61 (0.88)	2.39 (0.94)
	Post-test	2.10 (1.02)	2.44 (0.66)	2.41 (0.96)	2.32 (0.87)	2.50 (0.87)	3.13 (0.59)	3.36 (0.60)	2.98 (0.78)
	All	2.16 [0.31]	2.48 [0.31]	2.33 [0.28]	2.32 [0.18]	2.40 [0.23]	2.69 [0.24]	2.99 [0.24]	2.69 [0.14]
Total mood	Pre-test	99.40 (3.15)	98.86 (3.21)	98.48 (5.00)	99.26 (3.84)	97.10 (2.98)	99.51 (3.83)	97.88 (3.85)	98.12 (3.57)
disturbance	Post-test	100.67 (4.26)	100.01 (3.93)	98.20 (5.52)	99.52 (4.64)	96.78 (2.19)	96.80 (2.09)	96.20 (4.03)	96.60 (2.79)
	All	100.04 [1.47]	99.43 [1.47]	98.84 [1.32]	99.44 [0.82]	96.94 [0.95]	98.15 [1.01]	97.04 [1.01]	97.38 [0.57]

#### Effects of Time Interval on Walking

The analyses indicated a significant main effect of Time Interval on perceived exertion, heart rate, and self-selected gait speed (see Table 1), while no changes in mood states were recorded after the Experimental Phase as compared to the Pre-Experiment Phase (see Table 3). *Post hoc* comparisons indicated that the levels of perceived effort, heart rate, and self-selected gait speed increased significantly over the time intervals (see Table 4). Specifically, for gait speed, the value of the first interval was lower than the values of the subsequent intervals (*p*s ≤ 0.001), and the value of the second interval was lower than the values of the third and fourth ones (*p*s ≤ 0.01). For heart rate, the value of the first interval was lower than the values of the third and fourth ones (*p*s = 0.001), and the values of the second and third intervals were lower than those of the fourth interval (*p*s ≤ 0.05). The perceived exertion was lower for the first and second intervals as compared to the third and fourth ones (*p*s ≤ 0.02), and the values increased significantly between the third and fourth intervals (*p* = 0.004). Taken together, these findings are consistent with the results of a recent study showing that perception of effort and heart rate values increased over time during a 20-min self-selected exercise (Smith et al., 2015). Such increases would be due to the fact that individuals exercising at a self-selected pace would move progressively toward their ventilator threshold, i.e., the point separating aerobic from anaerobic metabolism (see Smith et al., 2015).

## Study 2

### Methods

#### Participants

Twenty-nine healthy female young adults (*M*_age_ = 22.86 years, *SD*_age_ = 3.49; *M*_height_ = 1.62 m, *SD*_height_ = 0.05; *M*_weight_ = 61.45 kg, *SD*_weight_ = 12.42) volunteered in Study 2 (green, *n* = 10; red, *n* = 10; white, *n* = 9). Participants in Study 2 were different than those who volunteered for Study 1. The final sample was composed of 28 participants (*M*_age_ = 22.96 years, *SD*_age_ = 3.51; *M*_height_ = 1.63 m, *SD*_height_ = 0.05; *M*_weight_ = 61.82 kg, *SD*_weight_ = 12.49) randomly assigned to one of the three experimental conditions (green, *n* = 10; red, *n* = 9; white, *n* = 9) as one participant reported an unsatisfactory score of perceived color typicality. Participants were asked to bring comfortable sports outfit and shoes for the experimental procedures, prior to which they signed a written informed consent in accordance to the Declaration of Helsinki. The experimental procedures were approved by the Institutional Review Board of Qatar University (IRB: QU-IRB 586-E/16).

#### Materials, Design, Procedure, Measures, and Analysis

The apparatus, experimental conditions, experimental procedures, measures, and analyses of this study were the exact same as the ones employed in Study 1. The unique specificity of Study 2 concerned the experimental task that consisted of running (instead of walking) for 20 min on the treadmill at the most comfortable speed.

### Results and Discussion

#### Preliminary Results

Results indicated no significant main effect of Colored Environment on perceived color typicality, body height, body weight, and leg length (see Tables 1, 2). As in Study 1, the preliminary results suggest that the subsequent findings may more likely be due to the experimental conditions than to differences between groups.

#### Effects of Colors

Although the analyses did not reveal a main effect of Colored Environment on any variables (see Tables 1, 3), they indicated a Colored Environment × Time Interval significant interaction effect on ratings perceived exertion (see Table 1). *Post hoc* comparisons indicated that perceived exertion was higher in the green environment as compared to the red environment at the fourth time interval, *p* = 0.03 (see Table 2 and Figure 1B). Moreover, in the green condition, values of perceived exertion increased over the time intervals: The perceived exertion values of the last two time intervals were higher than those of the first two time intervals, *p*s ≤ 0.004 (see Table 2). Our result seems contradictory to those of Akers et al. (2012) study showing that perceiving green while cycling at a moderate pace diminished the perception of effort. However, the participants in the study were asked to maintain a constant moderate level of cycling performance (i.e., 50% peak power output) over time (Akers et al., 2012), whereas the participants of the present study were asked to adjust periodically their running speeds so as to generate the most comfortable pace at any given moment. This suggests that although participants perceived their last interval of running as being more effortful in the green environment, as compared to other colored conditions, they preferred to maintain (and not reduce) their running intensity that was supposed to be subjectively perceived as the most comfortable. Indeed, many studies linked the psycho-physiological measure of perceived exertion with objective physiological measures of effort (e.g., heart rate) (Hampson et al., 2001; Faulkner and Eston, 2007) and considered it to act as a regulator of effort serving the energy conservation principle (e.g., St Clair Gibson et al., 2006; Gendolla and Richter, 2010; Majed et al., 2012; Silvestrini and Gendolla, 2013). Yet, in the present study, the lack of significant increase in heart rate (or intensity) could not directly explain the increase in perceived exertion in the last running interval of the green condition. We propose that psychological factors may have contributed to the choice of maintaining a running speed in a specific environmental setting (i.e., green environment), although perceived as more effortful. Previous studies suggested that preferred exercise intensity is related to the participants’ enjoyment and pleasure at that particular intensity (Parfitt et al., 2000). Therefore, the fact that participants “choose” to maintain a running speed that is perceived as more effortful (in the green condition) might be underpinned by a “need” to preserve a certain level of enjoyment or pleasure. This conclusion would merit further investigations to be better understand the role of psychological affective factors in regulating the self-selection of movement patterns.

The analyses also exhibited a Colored Environment × Experimental Phase interaction effect for tension (see Table 3). Specifically, the *post hoc* comparisons revealed that tension was lower after running for 20 min in the red environment as compared to the pre-experimental phase, *p* = 0.001 (see Table 4). Once again, our result sounds inconsistent with those of Akers et al. (2012) study showing that perceiving red while exercising at a moderate intensity generated more intense negative mood states (i.e., total mood disturbance and anger). Nonetheless, contrarily to what Akers et al. (2012) did, our study did not measure mood states during the ongoing experimental trial (but only before and after), thus limiting the comparison between results. However, and in line with the view that perceiving red may generate negative mood states and anxiety (e.g., Akers et al., 2012; Elliot and Maier, 2014; Recours and Briki, 2015), our finding may reflect a “relief” effect following the end of the exposure to red. Moreover, and consistently with the supposition that seeing red may hinder the optimization of the energy saving functioning, one might suggest that experiencing a decrease in tension after exercising during 20 min in a red (aversive) environment may reflect a state of fatigue. A recent research revealed that exposure to red, as compared to exposure to gray, enhanced distraction and decreased cognitive performances, when participants executed a supplementary cognitive task designed to deplete their self-control strength (Bertrams et al., 2015). Following that perspective, our results suggest that exposure to red while running for 20 min can deplete cognitive resources, thereby leading to the reduction of the intensity of subsequent emotions. Notwithstanding, such suppositions require further empirical investigations.

#### Effects of Time Interval on Running

The analyses showed a main effect of Time Interval for perceived effort and heart rate (marginal effect, *p* = 0.06) (see Table 1). For heart rate, the value of the first interval was lower than the value of the second one (*p* = 0.02) (see Table 2), while values of perceived exertion increased from the first and second intervals to the third and fourth ones (*p*s ≤ 0.03) (see Table 2). Those results support those found in Study 1. The analyses also revealed a main effect of Experimental Phase for tension (marginal effect, *p* = 0.05), fatigue, depression, esteem, vigor, confusion, and total mood disturbance (see Table 3), in the sense that positive mood states (i.e., vigor and esteem) appeared to increase from the pre-experiment phase to the recovery phase, whereas most of the negative mood states (i.e., tension, depression, confusion, and total mood disturbance) decreased (see Table 4). The only negative mood state that increased was fatigue (see Table 4), consistently with our previous finding regarding the increase in perceived effort over time. Generally, results on mood-related variables suggest that running for 20 min at a self-selected intensity can have positive implications on affective outcomes, supporting several studies that reported beneficial effects of most forms of aerobic exercise on mood and well-being. For example, Hansen et al. (2001) found that only 10 min of aerobic exercise (i.e., cycling at 60% of estimated VO_2_ max) were sufficient to improve vigor, fatigue, and confusion after only 20 min of exercise. An increasing number of studies report greater affective responses when the exercise intensity is self-selected as compared to low, moderate or high intensities (e.g., see Ekkekakis, 2009). Generally, our results corroborate the widespread belief of a positive acute effect of self-selected exercise on feelings and well-being.

## General Discussion

The present research aimed at examining whether and to what extent perceptions of green and red environments could affect people’s perceptions of effort, heart rate, affect and self-selected walking (Study 1) and running (Study 2) speeds when no performance goal was imposed. We hypothesized that exposure to green environment would foster adaptive psychobiological functioning and serve the action of the energy-saving principle in both walking and running, whereas we expected to find opposite effects in response to exposure to red.

Study 1 revealed that exposure to green when walking, as compared to exposure to red and white, was related to a decreased heart rate, whereas gait speed was not affected by the color condition. Gendolla and colleagues proposed that mental or physical effort mobilization would be driven by a principle of energy-conservation (Gendolla and Richter, 2010), and evidenced a positive relationship between the degree of task demand and the level of cardio-vascular reactivity (e.g., Brinkmann and Gendolla, 2008; Silvestrini and Gendolla, 2013). The result of Study 1 goes in line with our hypothesis that green might be processed as a calm, relaxing, and non-aversive (inoffensive) environment, thus inducing a decrease in participants’ cardio-vascular reactivity. Interestingly, Study 2 did not confirm such a significant effect of perceiving green on heart rate values when the mode of progression was running rather than walking. A first observation that arises when comparing findings from both studies is that perceiving colors (i.e., green) might affect differently the organism’s responses (e.g., heart rate) according to the task being performed. At present, it is hard to confirm if the inconsistent effect of perceiving green on heart rate is related to the mode of progression (i.e., nature of the task) or to the natural discrepancy in the intensities between walking and running. Even though participants were instructed to choose the most comfortable speed, running was naturally performed at higher intensities and associated with higher heart rate, perceived exertion and arousal levels (Table 2). These findings seem to indicate that seeing green might more greatly affect the less-demanding motor skill (i.e., walking), where participants are thought to be more sensitive (or less distracted) to information arising from the environment.

Nevertheless, it is worth mentioning that an unexpected increase in the levels of perceived effort was found at the last running interval only when exposed to the green environment, while no changes in the self-selected running speed or heart rate values were recorded across the color conditions. Such unexpected (or even counterintuitive) result goes against our initial hypotheses. According to the central governor model (e.g., St Clair Gibson and Noakes, 2004) and the motivational intensity theory (e.g., Wright, 1996) perceptions of effort and threat (e.g., tension, anxiety) would be essential to the regulation of behaviors while exercising. More specifically, perception of effort is regarded as a means to reduce (or even stop) the effort either when the brain engages protective actions while exercising–in response to the anticipation of a threat or danger that may harm the organism (e.g., hot and wet climate; prediction of the central governor model)–or when the exerciser is experiencing a lack of control causing anxiety and/or resignation (prediction of the motivational intensity theory). Taken together, such models emphasize on the capacity of perceived effort and anxiety to regulate effort for the sake of the preservation of the organism and, thus, for serving the principle of energy conservation. The greater levels of perception of effort at the end of the 20-min running trial in the green condition paralleled to undifferentiated levels of gait speeds across the experimental conditions may reflect the attempt from the human organism to maintain a certain level of optimal psychological/affective outcomes.

In sum, the results of both studies suggest that seeing green may promote energy conservation in walking–a low-demanding task–by slowing down heart rate, while in running–a moderately-demanding task–the effects of green color are not directly visible on the tested physiological parameter, where psychological factors seem better explained movement regulation.

### Benefits of Self-Selected Exercise

Study 1 revealed that a 20-min walking enhanced vigor, while Study 2 indicated that running for a same duration enhanced positive mood states (i.e., vigor and esteem) and reduced negative mood states (i.e., tension, depression, confusion, and total mood disturbance). These results indicate that both types of volitional exercise performed under comfortable conditions have positive implications on mood states, even though a 20-min running trial appeared to be more beneficial than walking for the same duration in healthy young adults. Our results confirm those of other studies evidencing the beneficial impact of volitional and self-selected walking and running patterns on emotions, moods, and self-perceptions (e.g., Ekkekakis, 2009; Kinnafick and Thøgersen-Ntoumani, 2014). Generally, our results support the results of studies that emphasized the fruitful effects of autonomous motivation toward exercise (i.e., regulation steered by a strong sense of volition and willingness) for quality of life, body satisfaction, physical self-esteem, and subjective well-being (e.g., Gillison et al., 2006, 2011; Thøgersen-Ntoumani and Ntoumanis, 2006; Sebire et al., 2009; Briki, 2016).

## Conclusion and Perspectives

The present research is the first to examine whether and how exposure to green vs. red environment could influence affective judgments, perceptions of effort, heart rate, and self-selected gait speeds while walking and running. Its most provocative result is that exposure to green, relative to other colors, slowed down heart rate in walking and increased perceived exertion after 15 min of running while self-selected gait speed was unchanged across all conditions. Based on different frameworks (e.g., governor central model, motivational intensity theory), we supposed that such effects might reflect the organism’s tendency to optimize certain parameters (e.g., affective judgments). Notwithstanding, this research comprises limitations. Chiefs among them are the laboratory/indoor nature of the manipulated environments and the absence of objective control of colors diffused by the TV screens. Firstly, the manipulation of the green environment in the laboratory setting was supposed to reproduce the visual stimuli associated with the green nature. However, contrarily to what Akers et al. (2012) did, we did not include any nature-related cues (e.g., drawn trees and forest). Hence, we believe that such a “denatured” experimental design could have lessened the effect of seeing green on our dependent variables (see Focht, 2009). Additionally, exercise under controlled laboratory environments is different from daily exercise in natural outdoor environments. Secondly, we did not use a spectrophotometer to control *a posteriori* the characteristics–in terms of hue, saturation and value–of the diffused colors by the screens. We only set such characteristics *a priori* and attempted to reduce such a limitation by controlling perceived color typicality (Elliot and Maier, 2014). Thirdly, we used small sample sizes that could have lowered statistical power and thus the chance of obtaining effects that are genuinely true (Button et al., 2013). Fourthly, this research did not take into account cultural and gender-related meanings of colors. For example, in the present research, all participants were Arabs and Muslims, and everybody living in any Arab and Muslim-majority countries know that the color green is sacred and symbolizes the paradise and, thus, can be associated with positive and appetitive meanings. As a result, further studies should either control or investigate the effects of personal, cultural, or gender-related meanings of and attitudes toward colors. Finally, given all of these limitations, the generalization of our results is limited.

To conclude, this research incites to pursue the examination of the effects of self-selected exercise, executed under green and red environments, on psychological, physiological, and behavioral parameters. However, future studies should take into account the above-mentioned limitations and further explore the luminosity criterion and possible differences in the effects of exposure to a specific given color versus a preferred one that can hold different personal/cultural meanings. For example, they could use sophisticated 3D virtual environments, attempting to reproduce natural outdoor settings while controlling input and output manipulated colors and luminosity. Other studies could even be based on real green environments (see Gladwell et al., 2013).

## Author Contributions

WB and LM conceived the study, drafted the manuscript, read, revised and approved the submitted version. Both authors organized the database and performed the data analysis and interpretations of the results.

## Conflict of Interest Statement

The authors declare that the research was conducted in the absence of any commercial or financial relationships that could be construed as a potential conflict of interest.
